# Highly potent dUTPase inhibition by a bacterial repressor protein reveals a novel mechanism for gene expression control

**DOI:** 10.1093/nar/gku882

**Published:** 2014-10-01

**Authors:** Judit E. Szabó, Veronika Németh, Veronika Papp-Kádár, Kinga Nyíri, Ibolya Leveles, Ábris Á. Bendes, Imre Zagyva, Gergely Róna, Hajnalka L. Pálinkás, Balázs Besztercei, Olivér Ozohanics, Károly Vékey, Károly Liliom, Judit Tóth, Beáta G. Vértessy

**Affiliations:** 1 Institutes of Enzymology and Organic Chemistry, RCNS, Hungarian Academy of Sciences, Budapest, Hungary; 2 Department of Applied Biotechnology and Food Sciences, Budapest University of Technology and Economics, Budapest, Hungary; 3 Doctoral School of Multidisciplinary Medical Science, University of Szeged, Szeged, Hungary

## Abstract

Transfer of phage-related pathogenicity islands of *Staphylococcus aureus* (SaPI-s) was recently reported to be activated by helper phage dUTPases. This is a novel function for dUTPases otherwise involved in preservation of genomic integrity by sanitizing the dNTP pool. Here we investigated the molecular mechanism of the dUTPase-induced gene expression control using direct techniques. The expression of SaPI transfer initiating proteins is repressed by proteins called Stl. We found that Φ11 helper phage dUTPase eliminates SaPIbov1 Stl binding to its cognate DNA by binding tightly to Stl protein. We also show that dUTPase enzymatic activity is strongly inhibited in the dUTPase:Stl complex and that the dUTPase:dUTP complex is inaccessible to the Stl repressor. Our results disprove the previously proposed G-protein-like mechanism of SaPI transfer activation. We propose that the transfer only occurs if dUTP is cleared from the nucleotide pool, a condition promoting genomic stability of the virulence elements.

## INTRODUCTION

*Staphylococcus aureus* (*S. aureus*) is one of the most important opportunistic pathogens causing nosocomial and community acquired infections, including several toxinoses, such as food poisoning, toxic shock syndrome (TSS), necrotizing pneumonitis and necrotizing fasciitis. Mobile genetic elements of *S. aureus* contribute largely to pathogenesis and to the spread of virulence factors and antibiotic resistance (1,2).

Major superantigenes (e.g. TSS toxin 1 (TSST-1), Enterotoxin B (SEB)) responsible for the different toxinoses are encoded as accessory genes by phage-related *S. aureus* pathogenicity islands (SaPIs) of diverse size (2–17 kb). SaPIs themselves do not encode any machinery for horizontal gene transfer, they take advantage of phage reproduction instead (2). In the absence of a helper phage, the expression of SaPI-encoded transfer initiating proteins (integrase and excisionase (3)) is repressed by SaPI-encoded repressor proteins called Stl. Helper phage infection or prophage activation relieves Stl repression and leads to the excision and extensive replication of SaPI. The resulting SaPI DNA is packaged into phage capsids (2). The helper phage proteins responsible for the de-repression are identified only in a few cases: SaPI1 is de-repressed by Sri, a DNA-binding protein, Sapibov2 is de-repressed by a small protein of unknown function, while SaPIbov5 and SaPIbov1 are de-repressed by dUTPases from phage 80α (for both) and phage Φ11 (for SapiBov1)) (4,5). In the latter case, it was shown also that phage Φ11 dUTPase disrupts the preformed Stl-DNA interaction, relieving the transcription of the repressed protein responsible for the initiation of the transfer (5).

The discovery of new ‘moonlighting’ functions of metabolic enzymes in gene expression regulation is of much current interest. In this specific case, dUTPase, a well characterized enzyme in pyrimidine biosynthesis and genome integrity maintenance, was found to regulate the transfer of mobile genetic elements. dUTPase is responsible for hydrolyzing dUTP, thereby providing dUMP and regulating the cellular dUTP: dTTP ratio (6–10).

A recent study showed that dUTPase mutants that are defective in dUTPase activity are also defective in SaPI activation (4). Based on indirect cellular experiments and the crystal structures of wild type and mutant phage dUTPases in complex with a dUTP analog, the authors also suggested that a specific conformational shift of the C-terminal arm of dUTPase, induced by dUTP binding is indispensable for the dUTPase:Stl interaction (4). The conformational shift of the C-terminal segment of trimeric dUTPases (such as dUTPases in phages 80α and Φ11) has been characterized in-depth in the literature as the single major conformational change occurring upon substrate binding and required for efficient catalysis (11–14). The dUTPase-regulated gene transfer was further proposed to adopt a mechanism highly reminiscent of G protein-mediated signaling, where the switching conformational change occurs upon GTP binding to the G protein (4). However, such a mechanism is in disagreement with the kinetic properties of the dUTPase enzyme cycle, which is fundamentally different from that of G proteins (15–20).

To resolve this contradiction, we aimed at a quantitative in-depth characterization of the dUTPase-induced de-repression mechanism. Our results from numerous biophysical methods disprove the previously suggested G protein-like mechanism and suggest an alternative regulation model that fits into a broad physiological context, as well.

## MATERIALS AND METHODS

### Cloning, protein expression and purification

Stl_SaPIbov1_ protein (GenBank ID AAG29617.1) supplemented with an N-terminal HIS-tag was cloned into the pGEX-4T-1 vector to allow glutathione-S-transferase fusion expression and purification (details are given in the Supplementary Material). In this study we used tag-free Φ11 dUTPases, that were expressed from pETDuet-1 (Novagen) vector as was described previously for Φ11DUT^WT^ (21). Purification was performed on a Q-sepharose ion-exchange chromatography, followed by gel filtration on a Superdex 75 column (GE Healthcare) using an AKTA Explorer purifier. For purification details see the Supplementary Material. Protein concentrations are given in monomers.

### Isothermal titration calorimetry (ITC)

ITC experiments were carried out at 293 K on a Microcal ITC_200_ instrument. Proteins were dialyzed into 20 mM HEPES (pH = 7.5), 300 mM NaCl, 5 mM MgCl_2_, 1 mM TCEP and were used at 36 μM (Stl, in the cell) and 230 μM (Φ11dUTPase^WT^, in the syringe) concentration. Both protein concentrations correspond to subunits. As a control, Φ11 dUTPase was also injected into the buffer to allow for considering mixing and dilution heat effects. The binding isotherms were fitted with an independent binding sites model ‘One Set of Sites’ (ORIGIN 7.5 software Microcal). This model is appropriate for any number of sites *n* if all sites have the same *K* and Δ*H*.

### Native gel electrophoresis

Native gel electrophoresis was performed in 8% polyacrylamide gels. After 2 h pre-electrophoresis with constant voltages of 100 V, the electrophoresis was performed for 2.5 h at 150 V in pH 8.7 Tris-HCl buffer. During electrophoresis the apparatus was cooled on ice. Note that 10 μl of a sample was added to each well. The gel was stained with Coomassie-Brilliant Blue dye.

### Quartz crystal microbalance (QCM) measurements

Stl was immobilized on sensor chips (Attana AB, Stockholm) (for details see the Supplementary Material). Binding experiments were performed with a continuous flow (25 μl/min) of running buffer (10 mM HEPES, 150 mM NaCl, 0.005% Tween 20, pH 7.4) allowing for a contact time of 90 s. Analyte samples were prepared in running buffer for Φ11 dUTPase^WT^ and Φ11 dUTPase^F164W^ (0.46 μM) in the absence and presence of 0.5 mM dUTP or 2 mM dUMP at 298 K. In the case of measurements with dUTP care was taken to ensure steady-state dUTP hydrolysis state during the experiment. The frequency response curves were analyzed by the BIAevaluation 4.1 software.

### Steady-state fluorescent measurements

For steady-state measurements of Trp fluorescence a Perkin Elmer EnSpire Multimode Plate Reader was used (details in Supplementary Material). For titration the binding partner was pre-incubated in assay buffer (phosphate buffered saline (PBS) (pH 7.3), 5 mM MgCl_2_, 400 mM NaCl) for 20 min. Titration results were fitted to the quadratic binding equation describing 1:1 stoichiometry for the dissociation equilibrium with no cooperativity:

(1) \begin{equation*} y = s + \frac{{A\left[ {(c + x + K) - \sqrt {(c + x + K)^2 - 4cx} } \right]}}{{2c}}, \end{equation*}

where *x* is the concentration of titrant and *y* is the fluorescence intensity, *s* = *y* at *x* = 0, *A* is the total amplitude of the fluorescence intensity change, *c* is the enzyme concentration, *K* is the half-saturation coefficient. The concentrations of titrands are given in the figure legends. All measurements were done at 293 K.

### Transient kinetics experiments

Stopped-flow measurements were carried out using an SX-20 (Applied Photophysics, UK) stopped-flow instrument, following Trp fluorescence at 293 K, as described previously (17,18). Typically 5–8 traces were collected and averaged. The mixed species and their concentrations (post-mixing) are indicated in the figure legends.

### Enzyme activity assay

Proton release during the transformation of dUTP into dUMP and PPi was followed continuously at 559 nm at 293 K (19) using a JASCO-V550 spectrophotometer. Reaction mixtures contained 10 nM enzyme and varying concentrations of Stl in activity buffer (1 mM Hepes (pH 7.5), 5 mM MgCl_2_, 150 mM KCl and 40 μM Phenol Red indicator). The reaction was started with the addition of 30 μM dUTP after 5 min pre-incubation of the two proteins. Initial velocity was determined from the slope of the first 10% of the progress curve.

### Electrophoretic mobility shift assay (EMSA)

EMSA experiments were done using an 183mer oligonucleotide (Stl binding site_183_) derived from the 171mer oligonucleotide described previously (5). Stl binding site_183_ (75 ng) and the investigated proteins were mixed in EMSA buffer (PBS (pH 7.3), 5 mM MgCl_2_, 75 mM NaCl, 0.5 mM ethylenediaminetetraacetic acid) in the presence or absence of α,β-imido-dUTP (dUPNPP) in 20 μl total volume. Before loading onto 8% polyacrylamide gel the samples were incubated for 15 min at room temperature. Electrophoresis was performed in Tris- Borate- EDTA (TBE) buffer for about 60 min at room temperature, after 1 h pre-electrophoresis. Gels were detected with a Uvi-Tec gel-documentation system (Cleaver Scientific Ltd., Rugby, UK) using GelRed staining (Biotium).

### *S. aureus* genome analysis

Completed genomes (to date 03/05/2014; http://www.ncbi.nlm.nih.gov/genome/genomes/154) of different *S. aureus* strains were searched in the REFSEQ database with trimeric dUTPase (Φ11 dUTPase, GeneID: 1258034) and with dimeric dUTPase (Φeta3 dUTPase, GeneID:927341) sequences using tblastn (http://blast.ncbi.nlm.nih.gov/Blast.cgi?PROGRAM=tblastn&PAGE_TYPE=BlastSearch&LINK_LOC=blasthome). The search was performed with the basic parameter settings offered by the software. Prophage regions were identified based on the publications describing the genomic sequence or by PHAST software (22).

## RESULTS AND DISCUSSION

### Complex formation between Stl and dUTPase

**Figure 1.**
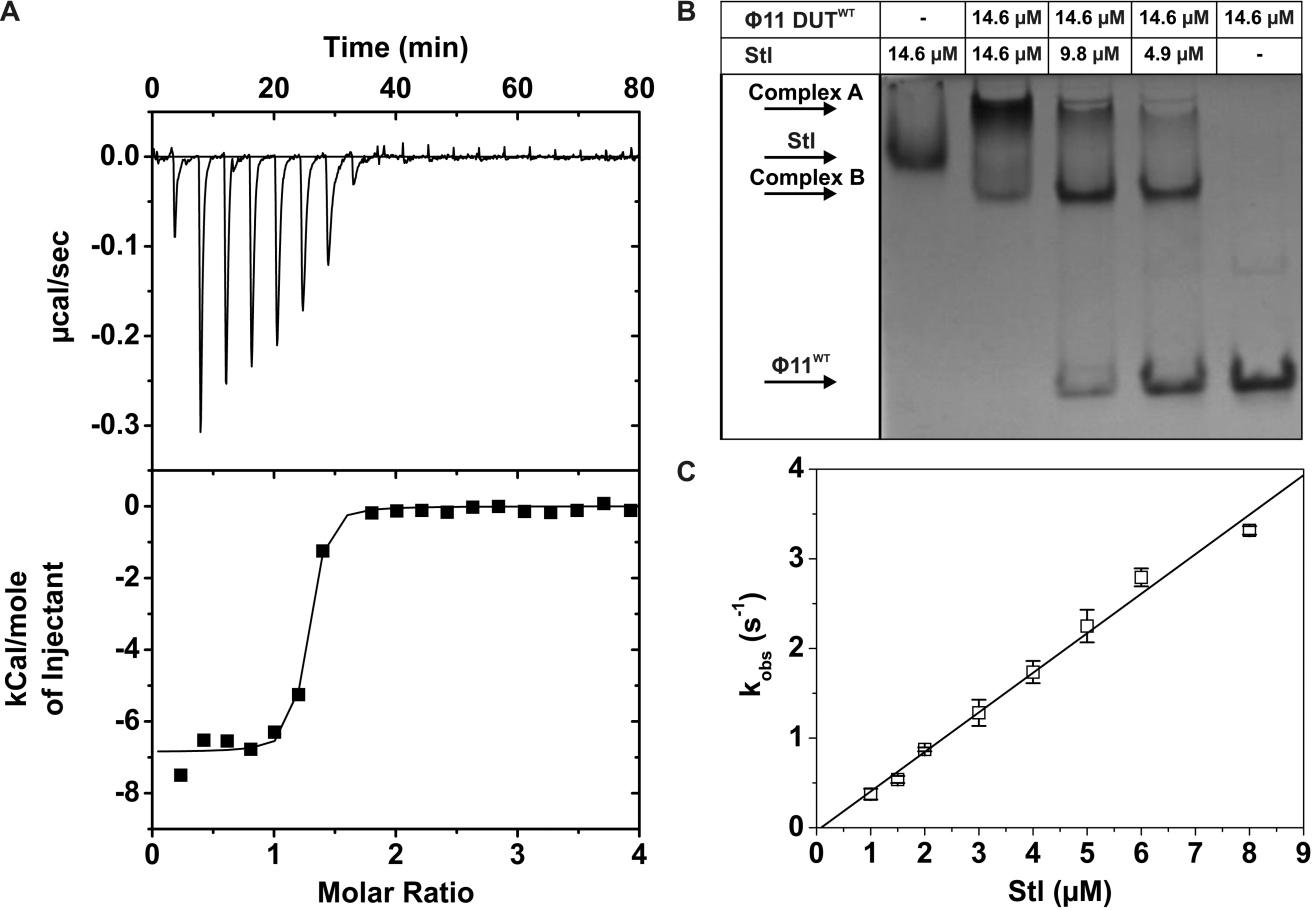
Φ11 dUTPaseWT and Stl form a tight complex with slow kinetics. **(A)** ITC measurement of dUTPase:Stl complex formation. The smooth line represents the fitted model, assuming one binding site. For the fitted parameters see Table 1. **(B)** Shows the result of native gel electrophoresis. Species and concentrations are indicated on the figure. **(C)** Shows the concentration dependence of the pseudo-first-order rate constant (*k*_obs_) observed upon Φ11 DUT^F164W^:Stl complex formation. Error bars represent SD for *n* = 2. Linear fit to the data (*r*^2^ = 0.99) yielded the association rate constant *k*_on_ = 0.41 ± 0.014 μM^−1^s^−1^. The *y* intercepts of the fitted line was too small for the exact determination of the *k*_off_ value. However, the *k*_off_ value is small and indicate submicromolar *K*_d_.

The physical interaction between Φ11 dUTPase and SaPIbov1 Stl was proposed to result in the release from Stl repression observed in cellular systems (5). However, no quantitative description of a dUTPase-Stl protein complex was available. To provide such data indispensable for mechanistic insights, we cloned and purified both protein components of the putative complex. ITC data indicated that the Φ11 dUTPase and Stl form a considerably strong complex (dissociation constant is 0.10 ± 0.03 μM) (Figure 1A, Table 1)). A variety of additional methods confirmed this complex equilibrium: native gel electrophoresis (Figure 1B), soft-ionization mass spectrometry (Supplementary Figure S1A and B) and size-exclusion chromatography (Supplementary Figure S1C). As seen in the native gel, at stoichiometric amounts of Stl and Φ11dUTPase (1:1 with respect to monomeric species or subunits), no band is observable at the positions of the free proteins, arguing that complexation is maximal at this concentration ratio (complex ‘A’ in Figure 1B). It is also evident that at substoichiometric amounts of Stl another complex form is observed (complex ‘B’ in Figure 1B), probably reflecting an altered composition within the heterooligomer of the two proteins (see also Supplementary Results and Discussion).

**Table 1.**
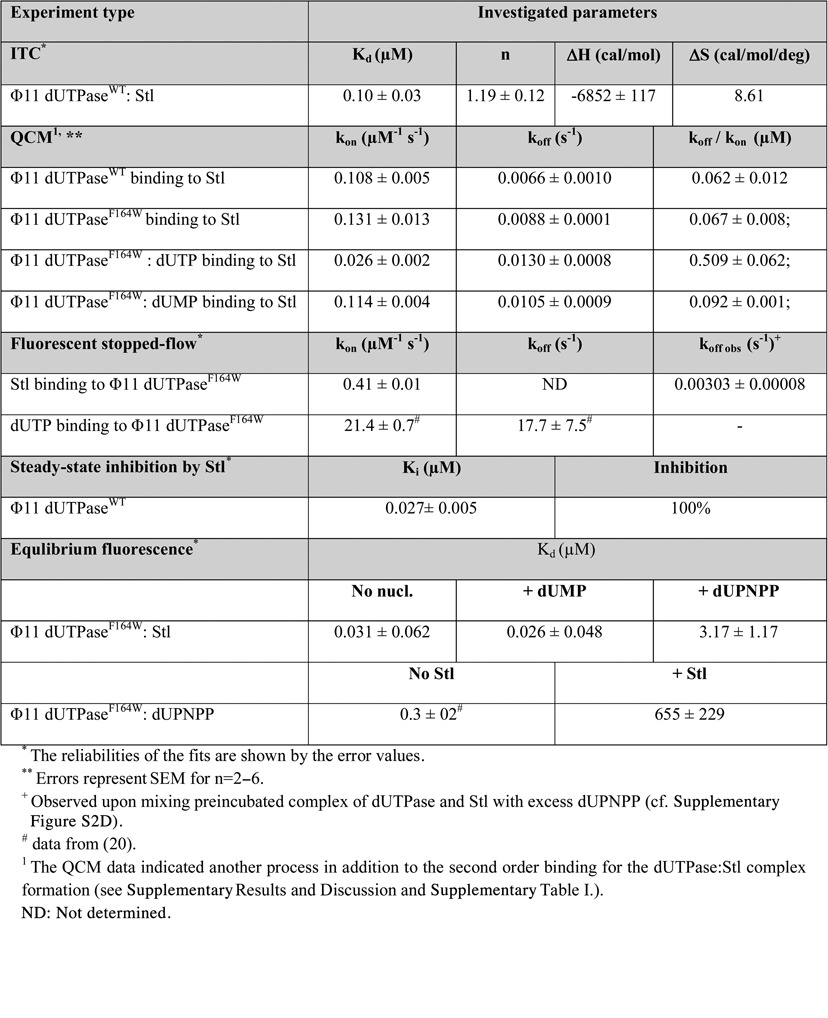
Kinetic and thermodynamic parameters of Φ11 dUTPase: Stl_SaPIbov1_ interaction in the presence and absence of uracil nucleotides

Kinetics of complex formation was analyzed by QCM and stopped-flow measurements. QCM results showed that both the association and dissociation rate constant of the dUTPase:Stl complexation are approximately two orders of magnitudes lower than those data for the dUTPase:dUTP complexation (Table 1, Supplementary Figure S1D, cf. also (18,20)). The equilibrium dissociation constants, calculated from the association and dissociation rate constants (*K*_d_ = *k*_off_/*k*_on_), is in good agreement with the ITC data (Table 1). The QCM data also indicate that dUTPase and Stl complex formation may involve a conformational change also, although this suggestion needs further experimental investigation (see Supplementary Results and Discussion and Supplementary Table S1).

The slow and tight binding character of the complex formation between Stl and dUTPase was also confirmed by fluorescent experiments (Figure 1C and Supplementary Figure S1E and F) exploiting the useful tryptophan label within the active site of dUTPase that does not change the enzymatic properties (Φ11 dUTPaseF164W (20)). We repeated the QCM experiments with the Φ11 dUTPaseF164W protein and Stl, and found that the measured parameters did not show any significant change as compared to the wild-type dUTPase (Table 1). Hence, we conclude that the Φ11 dUTPaseF164W shows wild-type behavior in both enzyme kinetics and Stl-interaction, allowing us to use this useful mutant in stopped-flow and other experiments as well. As shown on Supplementary Figure S1E, Stl binding to Φ11 dUTPaseF164W enhances the fluorescent intensity. Using this fluorescence intensity change to detect Stl binding to dUTPase (Supplementary Figure S1F) one binding step was observed that was identified as the bimolecular complex formation (Figure 1C). The rate constants yielded from these experiments are in good agreement with QCM data (Table 1).

Our experiments clearly indicate that a strong physical interaction takes place between dUTPase and Stl in the absence of dUTP. This finding does not support the earlier suggestion that this interaction require the presence of dUTP (4). To gain insight into how substrate and product (dUTP and dUMP) may modulate the dUTPase-Stl interaction, we performed further experiments.

### dUTPase:Stl complex formation abolishes the known physiological function of both proteins

**Figure 2.**
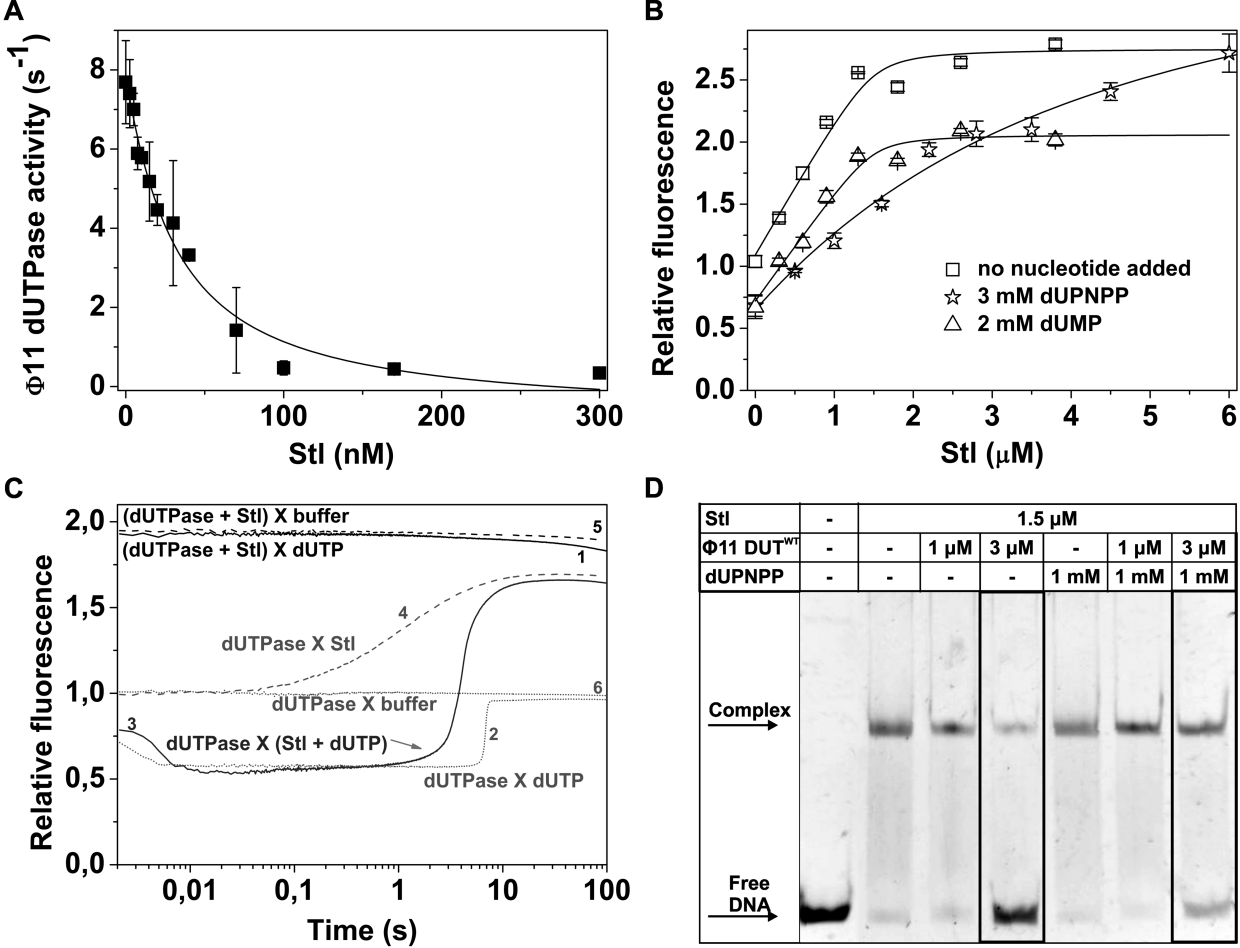
dUTPase:Stl complex formation eliminates the physiological function of both proteins. **(A)** Inhibitory effect of Stl on Φ11DUT^WT^ (10 nM) catalytic activity. Data represent average and error of three parallel measurements. Solid line represents fit of quadratic binding equation to the data, yielding IC_50_ 26.64 ± 5.07 nM. **(B)** Shows titration of Φ11DUT^F164W^ (1.5 μM) and Φ11DUT^F164W^ (1.5 μM): dUPNPP (3 mM)/dUMP (2 mM) complex with Stl. Error bars represents SD for *n* = 3. Solid lines represent quadratic fits to the data (see Equation (1)). Dissociation constants from the fitted model are shown in Table 1. **(C)** Shows transient kinetic investigation of the mixing order dependency of Stl inhibition. 2 μM d Φ11DUT^F164W^, 3 μM Stl and 50 μM dUTP was mixed (post-mixing concentrations: X indicates the mixing of species in syringe A and B (syringe A X syringe B), parenthesis indicates that the components were pre-mixed. The curves are shown from 0.002 s (after the dead time). **(D)** Effect of dUPNPP on dUTPase derepression activity characterized by EMSA.

We measured the enzymatic activity of dUTPase in the dUTPase:Stl complex and found that Stl exerts highly potent inhibition of dUTPase activity with an IC_50_ value that approximates the *K_d_* of the protein–protein complex (Figure 2A, Table 1). This inhibition is only observed if dUTPase is pre-incubated with Stl prior to dUTP addition. Such behavior is typical for a slow and tight binding inhibitor (23) and is in excellent agreement with data obtained for the formation of the dUTPase:Stl complex (Figure 1, Table 1) as well as with the previously published kinetics of dUTP binding (20).

Stl stands as the first and single potent and directly identified protein inhibitor of dUTPase. Earlier suggestions for Drosophila and phage PBS2 proteins remained elusive (24,25). The regulation of the uracil content of DNA primarily depends on dUTPase and on uracil-DNA glycosylases (UDG-s) (26–31). It is therefore relevant to note that a similarly tight binding protein inhibitor (UGI–Uracil Glycosylase Inhibitor) of the main UDG, UNG is encoded in phage PBS1 and PBS2 (32). Interestingly, UGI was shown to be capable of inhibiting UNG-s from other species as well (33). It remains to be seen if Stl may prove to be a general dUTPase inhibitor, as well.

In order to better understand the mechanism of the dUTPase:Stl interaction and its functional consequences, we investigated the binding of Stl to dUTPase in the presence of the substrate, dUTP (or the substrate analogue dUPNPP) and in the presence of the product, dUMP. Both in equilibrium fluorescence titration (Figure 2B and Supplementary Figure S2A) and QCM experiments (Supplementary Figure S2B) we found that the presence of dUTP or dUPNPP strongly interferes with Stl:dUTPase complex formation. The fluorescence titration of the dUTPase:dUPNPP complex with Stl (Figure 2B) resulted in an equlibrium fluorescence intensity that was identical to that of the dUTPase:Stl complex implying that Stl displaced all dUPNPP. Hence, Stl and dUPNPP compete for binding to dUTPase (cf. also limited proteolysis results reported in Supplementary Figure S2C). The presence of Stl in turn inhibited the formation of the dUTPase:dUPNPP complex (Supplementary Figure S2A). On the other hand, dUMP, the product of the dUTPase reaction, and Stl do not influence the binding of each other (Figure 2B). The formation of a dUTPase:dUMP:Stl ternary complex is indicated by a distinct fluorescence state characterized with lower fluorescence intensity than that of the dUTPase:Stl complex (Supplementary Figure S1E and Table 1).

Transient kinetic experiments also showed that pre-incubation of dUTPase and Stl fully prevented any enzymatic reaction on the time scale used to observe the reaction in the absence of Stl (Figure 2C, compare curves 1 and 2, cf. also with the controls (curves 5 and 6)). At longer time scales, a slow decrease in fluorescence intensity followed by a fluorescent increase, reminiscent of dUTP binding and product release (cf. (18,20)), was observed (Supplementary Figure S2D). In agreement with the competition between Stl and dUTP for dUTPase binding, single exponentional fit to decreasing phase yielded a dUTP concentration (500–2300 μM) independent *k*_obs_ = 0.00303 ± 0.00008 s^−1^, which is in agreement with the rate constant of Stl dissociation from dUTPase. We therefore propose that when dUTP is added to the pre-formed Stl:dUTPase complex, dUTP binding and hydrolysis requires Stl dissociation. On the other hand, if the mixture of dUTP and Stl are added together to dUTPase, the fluorescence time course (Figure 2C, curve 3) is analogous to the curve observed in the absence of Stl (curve 2) except that the equilibrium fluorescence intensity approaches that of the dUTPase:Stl complex (curve 4). Stl binding, reflected in fluorescence increase (paralleled with product release, that also causes fluorescence increase, cf. arrow on curve 3), may only occur when the concentration of the dUTPase:dUTP Michaelis complex starts to decrease. This is in agreement with the steady-state results and reinforces the conclusion that Stl is a competitive, slow and tight binding inhibitor of dUTPase.

Based on the direct experimental data of numerous independent assays (Figure 2A–C and Supplementary Figure S2), we suggest that dUTP and Stl compete for dUTPase binding and that the dUTPase:dUTP complex is inaccessible for Stl. Therefore, the previously suggested model stating that dUTP mediates the dUTPase:Stl interaction (4) remains unsubstantiated.

It was also of immediate interest whether the de-repression activity (i.e. the physiological function) of the dUTPase:Stl complex is also modulated by dUTP. To this end, we performed EMSA experiments (Figure 2D). We observed that dUTPase inhibits the binding of Stl to its cognate DNA sequence only in the absence of the dUTP analog. This suggests that dUTP counteracts the de-repression event by preventing dUTPase:Stl complex formation.

The EMSA results again disagree with the previous model in which dUTP was suggested to enhance de-repression and the ensuing horizontal transfer of mobile genetic elements. Our results support instead that dUTP counteracts de-repression. Another key point of the previous model concerned the role of the C-terminal arm of dUTPase: it was suggested, based on indirect experiments, that de-repression may only occur if the C-terminal arm of dUTPase adopts a predominantly ordered conformation as it does in the dUTP-bound form. The present direct EMSA experiments, however, clearly show that a C-terminal arm-truncated dUTPase may also disrupt Stl binding to DNA, very similarly to the wild type (Supplementary Figure S3). Hence, the dUTPase:Stl interaction does not seem to require the presence of the C-terminal arm.

### Staphyloccus aureus strains do not encode genomic dUTPase

To consider the physiological relevance of the regulatory role of dUTP, we need to take cellular nucleotide concentrations into account. It is known that the general cellular concentrations of dNTPs are in the order of 5–40 μM (34), with the exception of dUTP which is under control by dUTPase (35) and normally, its concentration is around 0.2 μM only (34). dUTPase is considered to be a ubiquitous enzyme, due to its important role in nucleotide pool control. Accordingly, knock down of dUTPase results in significant increase of the dUTP level gaining up to the level of the canonical dNTPs, as it was shown in several human cell lines (36–38). According to our results an elevated cellular dUTP concentration probably interferes with the dUTPase:Stl interaction and consequently inhibits the activation of SaPI transfer.

To investigate if dUTPase, the major regulator of dUTP levels, is also present in *S. aureus*, we analyzed the genome data available for different *S. aureus* strains. Interestingly, neither of these strains encode an endogenous dUTPase gene. However, in most cases the chromosome contained integrated prophages carrying dUTPase genes (Supplementary Table S2). Importantly, the expression of proteins located in the replication module of prophages are probably under repression in the lysogenic phase and dUTPase expression is upregulated only after prophage induction (39).

Such an expression pattern of dUTPase is expected to be paralleled with an increased dUTP level within *S. aureus*. Interestingly, it was also found recently that a conserved *S. aureus* protein (SaUGI) has an UNG inhibitory effect (40). Lack of dUTPase and UNG activity may lead to the accumulation of uracil in genomic DNA (26,41) and to an increased mutagenic rate in this biomedically challenging pathogenic microorganism (c.f. (30,42–43)).

### A novel mechanism for the dUTPase-regulated molecular switch

**Figure 3.**
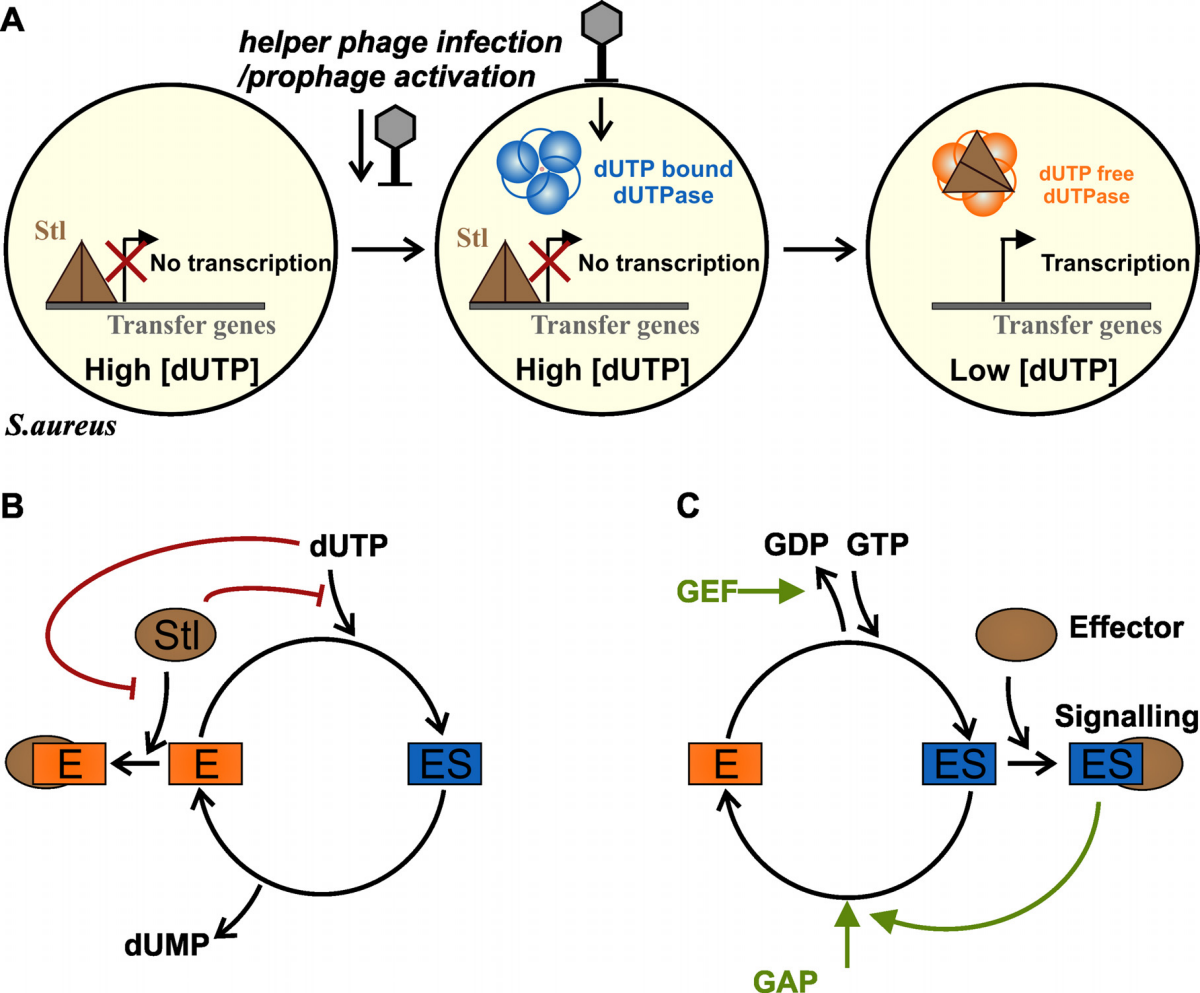
Model of the mechanism of dUTPase-controlled SaPI activation. **(A)** Shows our novel model for dUTPase-based SaPI activation. **(B)** Molecular mechanism of dUTP controlled dUTPase:Stl interaction. **(C)** Molecular mechanism of G-protein-based switch. On panels B and C ES represents substrate bound, while E represents substrate-free enzyme (free enzyme or product bound enzyme). Red and green arrows represent inhibition and activation, respectively.

Figure 3A shows our model for the regulation of horizontal gene transfer by dUTP. We propose that in the absence of genomic dUTPase, *S. aureus* strains may contain a relatively high dUTP concentration. Upon helper phage infection or prophage activation, phage dUTPase is expressed and hydrolyses dUTP in a fast and efficient process. dUTPase and Stl do not interact efficiently if dUTP is present, therefore, dUTPase becomes available for binding to the Stl repressor protein only after the dNTP pool is cleared from dUTP. In our proposed mechanism, the helper phage dUTPase breaks down dUTP and subsequently activates the transcription of the transfer initiating proteins within the pathogenicity island.

Our data leaves an earlier G protein-like hypothesis unsubstantiated (4). The fundamental differences between G protein regulation and the dUTPase:Stl interaction-based regulation are displayed in Figure 3B and C, and in Supplementary Table S3. dUTPases are responsible for fast and efficient clearance of dUTP from the cellular pool, facilitated by fast release of the products. dUTPases are predominantly dUTP-bound while the level of dUTP is high, and the hydrolysis product dUMP is quickly released (cf. (18,20)). G proteins, however, are very slow hydrolases and exist predominantly in ligand-bound states. For both hydrolysis and product release, G proteins require additional protein regulators (GAPs (G-protein Activating Protein) and GEFs (Guanoside Exchange Factor)). The multistep regulatory pattern relying on various factors allows G proteins to fulfill widespread finely tuned signaling processes. dUTPases, on the other hand, are simple and fast catalysts of dUTP cleavage.

For the dUTPase-dependent molecular switch, the dUTPase:Stl interaction is the only yet described example, and it remains to be seen if further such dUTPase-binding proteins may be identified. It is important to emphasize that while our model of the dUTP-regulated dUTPase:Stl interaction contradicts the earlier proposed G protein-like scheme, it is still fully consistent with the experimental observations reported in the same study (4), as demonstrated in Supplementary Table S4. Importantly, these *in vivo* results also show that the extent of SaPI activation correlates with dUTPase activity.

## CONCLUSION

We described a molecular mechanism that connects the regulation of gene expression to the regulation of the enzymatic activity of trimeric dUTPase, a nucleoside triphosphate hydrolase that is responsible for genome integrity. Our data show that dUTPase strongly binds to the Stl repressor protein in the absence of substrate and this complex disrupts the capability of Stl binding to its cognate DNA element. We also found that the presence of dUTP precludes Stl binding to dUTPase. Despite being considered to be ubiquitous, several *S. aureus* strains do not encode endogenous dUTPase, suggesting high intracellular dUTP level. We propose that helper phage dUTPases may be responsible for sanitizing the dUTP pool. Once dUTP is hydrolyzed, dUTPase switches function and becomes quantitatively available for driving the gene expression that initiates the horizontal transfer of SaPI. The countereffect of dUTP suggests that the excision and extensive replication of SaPI occurs under dUTP-cleaned, sanitized nucleotide pool conditions, ensuring uracil-free replication of the subsequently transferred mobile genetic element. The presence of uracil in SaPI DNA is probably unfavorable, as the uracil content of a mobile genetic element may negatively influence its integration into the DNA of the new host, as it was recently shown for HIV (44,45). In case of HIV, if the new host cell contains an active UNG, the uracilated viral DNA may be degraded before its integration into the genome could happen (44).

The presently discovered specific and efficient inhibition of dUTPase, not described before, will greatly contribute to the understanding of the communication between pathways responsible for maintaining nucleotide pools, DNA damage recognition, repair and genome integrity.

## SUPPLEMENTARY DATA

Supplementary Data are available at NAR Online.

SUPPLEMENTARY DATA
