# Supplementary material for: Highly potent dUTPase inhibition by a bacterial repressor protein reveals a novel mechanism for gene expression control
Source: Nucleic Acids Res. 2014 Oct 1;42(19):11912–20. doi: 10.1093/nar/gku882 (PMC4231751; doi:10.1093/nar/gku882)
Supplement: SUPPLEMENTARY DATA [file supp_42_19_11912__index.html]

Highly potent dUTPase inhibition by a bacterial repressor protein reveals a novel mechanism for gene expression control — Highly potent dUTPase inhibition by a bacterial repressor protein reveals a novel mechanism for gene expression control — SUPPLEMENTARY DATA 

# Highly potent dUTPase inhibition by a bacterial repressor protein reveals a novel mechanism for gene expression control

## SUPPLEMENTARY DATA

**Files in this Data Supplement:**

- SUPPLEMENTARY DATA
