# Supplementary material for: Highly potent dUTPase inhibition by a bacterial repressor protein reveals a novel mechanism for gene expression control
Source: Nucleic Acids Res. 2014 Oct 1;42(19):11912–20. doi: 10.1093/nar/gku882 (PMC4231751; doi:10.1093/nar/gku882)
Supplement: SUPPLEMENTARY DATA [file supp_gku882_nar-01869-h-2014-File005.docx]

# Highly potent dUTPase inhibition by a bacterial repressor protein reveals a novel mechanism for gene expression control

# Running title: dUTP level controls horizontal gene transfer

Judit E. Szabó^1^*, Veronika Németh^1^, Veronika Papp-Kádár^1^, Kinga Nyíri1, Ibolya Leveles^1^, Ábris Á. Bendes^1^, Imre Zagyva^1^, Gergely Róna^1^, Hajnalka Pálinkás^1^, Balázs Besztercei^1^, Olivér Ozohanics ^1^, Károly Vékey^1^, Károly Liliom^1^, Judit Tóth^1^* and Beáta G. Vértessy^1,2^*

^1^ Institutes of Enzymology and Organic Chemistry, RCNS, Hungarian Academy of Sciences

^2^ Dept. Biotechnology, Budapest University of Technology and Economics

* To whom correspondence should be addressed. Beáta G. Vértessy (Email: [vertessy.beata@ttk.mta.hu](mailto:vertessy.beata@ttk.mta.hu)) Correspondence may also be addressed to Judit Tóth (Email: [toth.judit@ttk.mta.hu](mailto:toth.judit@ttk.mta.hu)) and Judit E. Szabó (Email: [szabo.judit.eszter@ttk.mta.hu](mailto:szabo.judit.eszter@ttk.mta.hu))

## Supplemental Results and Discussion

#### Oligomerization of Stl

The electrospray mass spectrum of Stl presented in (Suppl. Fig. 1A) shows the presence of two abundant species in the 1500-3500 and in the 3500-6000 m/z range. Molecular mass of these species are 32910 ±40 Da and 65860 ±80 Da, corresponding to the monomer (S_1_) and dimer (S_2_) form of Stl, respectively (in good agreement with the monomer molecular mass of 32,896 calculated from the amino acid sequence (http://web.expasy.org/protparam/). Based on the integrated peak intensities the S_1_ : S_2_ratio is 10:9 (rough estimation, due to the overlapping peaks). Other oligomers were not detected. Size-exclusion chromatography (Suppl. Fig. 1C) also showed that Stl exists in an equilibrium between monomer and dimer forms. The oligomeric status of Stl has never been addressed before. However, dimerization of DNA binding proteins is a frequently occurring phenomenon.

#### Stochiometry of dUTPase Stl complex

The electrospray mass spectrum of Φ11 dUTPase^WT^: Stl mixture presented in Suppl. Fig. 1B shows the existence of different species in the sample. Below 3600 m/z different various charge states of the of Φ11 dUTPase^WT^ monomer (Φ_1)_ are visible in low amount, while in the range of 3700 – 5000 m/z the trimer form (Φ_3_) of Φ11 dUTPase^WT^ is observable with low intensity (59660 ±20 Da), as also described in (1). Note, that the dimer form of dUTPase (Φ_2_) is not observed – this strongly suggests that the well-observable Φ_3_ form is indeed the stable, native complex. An abundant species in the 4800-6000 m/z range is observable. The molecular mass of this species (125500 ±80 Da) corresponds to the Φ_3_S_2_ complex (consisting of Φ_3_ and S_2_ or 2 * S_1_). Free Stl molecules (S_2_ or S_1_) and other complex forms were not observed in this mass/charge range. As shown by native gel results Suppl. Fig. 1B Φ11 dUTPase^WT^ and Stl form more types of complexes. Based on the mass spectrometry (MS) and analytical gel filtration Stl is in equilibrium between monomers and dimers (see Suppl. Fig. 1B and suppl. Fig 1C), while Φ11 dUTPase^WT^ is present as a trimer (Φ_3_) at least in 90% (1). Therefore it is straightforward to assume that the different species are the complexes of Φ_3_ plus different number of Stl molecules (monomers and dimers). If Stl and Φ11 dUTPase^WT^ are in stoichiometric concentration neither the distinct band of free Φ11 dUTPase^WT^ nor the distinct band of free Stl are observable (see Suppl. Fig. 1B lane 4). Therefore complex A is probably equivalent to the Φ_3_S_3_ complex consisting of Φ_3_ and three Stl molecules (3 * S_1_ or S_2_ + S_1_). If the concentration of Stl is substoichiometric to Φ11 dUTPase^WT^ (see Suppl. Fig. 1B lane 2 and 3) another complex species, complex B also appears. The appearance of complex B at substoichiometric Stl concentrations indicates that this complex may contain Φ_3_ and fewer than 3 Stl molecules. If this complex consisted of Φ_3_ and one S_1_, then at 3:1 Φ11 dUTPase^WT^: Stl ratio (see Suppl. Fig. 1B B lane 2) the concentration of complex forming species would be stoichiometric and neither free Stl nor free Φ11 dUTPase^WT^ would be present (used protein concentrations were high above the K_d_ of complex formation). However free Φ11 dUTPase^WT^ is present, indicating that complex B corresponds to the Φ_3_S_2_ complex (consisting of Φ_3_ and S_2_ or 2 * S_1_), observed also by MS.

In MS we could not observe the Φ_3_S_3_ complex. Assuming that this complex exists (based on native gel), there are two different explanation for the lack of this complex in MS: *i,* the stabilities of the different complexes are different under the different conditions of native gel and MS; *ii,* Stl is present in substoichiometric concentration in MS due to stability problems in lack of high salt concentration. Since Φ11 dUTPase^WT^ and Stl were mixed stoichiometrically in the MS measurement, the absence of free Stl species from the mass spectra argues for the second explanation.

#### Kinetics of the complex formation between dUTPase and Stl

The kinetics of the dUTPase-Stl interaction was investigated by two independent methods. One of the methods, Quartz Crystal Microbalance (QCM) yielded the best fit to the data when a two state reaction model was applied (Suppl. Fig. 1D, Table I and Suppl. Table I). This model assumes bimolecular complex formation, followed by a conformational change. It is important to highlight that QCM is actually refractory to conformational changes, since it only reflects mass changes. However, according to the manufacturer’s manual, a good fit of experimental data to a multi-state model might be taken as an indication for a putative conformational change. Using the other, fluorescence detection-based method however, we did not find evidence for a conformational change within the Φ11 dUTPase:Stl complexes. The K_d_ values calculated from the k_off_/ k_on_ ratio, determined in the QCM experiments, are in good agreement with the K_d_ values obtained by equlibrium methods, therefore only the kinetic properties of bimolecular complex formation were analyzed in detail (see Table I). The data on the putative conformational change observed by QCM are summarized in Suppl. Table I. The two state reaction model fitted to the QCM data may be the result of the formation of more than one type of Φ11 dUTPase:Stl complex. To elucidate this issue, a further detailed kinetic analysis of the complex formation is necessary, that will be the subject of another paper.

## Supplemental Experimental Procedures

**Reagents**

Molecular biology products were from New England Biolabs (US) and Fermentas (Canada), electrophoresis and chromatography reagents were from Bio-Rad (US) and Qiagen (Netherland). Phenol red was from Merck (Germany), dUDP and dUPNPP were from Jena Bioscience (Germany), dUTP and other chemicals were from Sigma-Aldrich (US), if not indicated otherwise

### Cloning of Stl

The cDNA of the Stl_SaPIbov1_ protein (GenBank ID AAG29617.1) from *Staphylococcus aureus* was synthesized as a codon-optimized (EnCor Biotechnology Inc.) construct. The codon-optimized construct was cloned into the vector pETDuet-1 from Novagen with EcoRI and NotI restriction sites using the services of Eurofins MWG Operon. Thus, a His-tag and a thrombin cleavage site were attached to the N-terminal protein sequence. For glutathione-S-transferase (GST) fusion expression, Stl was further amplified from this expression vector, and was cloned into the EcoRI/XhoI restriction sites of the pGEX-4T-1 vector in frame with the N-terminal GST tag, as well as with a thrombin cleavage site and a 6xHis tag between the GST and the dUTPase sequence. For the amplification of Stl the Stl_pETDuet-1_-F (5’-TATTGAATTCCATCATCATCATCATCACGGCAGCATGGAAGGCGCGGGCCAGATG-3’) and the Stl_pETDuet-1_-R (5’-GGTCCTCGAGTTAGTTGGTATCTTTTTCCAGAATAATTTTTTTCTGATG-3’) primers were used. The resulting constructs were verified by DNA sequencing at Eurofins MWG Operon.

### Expression of Stl

For expression of Stl,cells transformed with respective plasmids were propagated in 500 ml LB till exponential growth, then the culture was cooled to 303 K and then induced with 0.5 mM iso-propyl-β-D-thiogalactoside. After induction, the cell cultures were grown for a further 4 h at 293 K. Finally the cells were harvested by centrifugation and stored at 193 K. Subsequent manipulations were carried out on ice.

### Purification of Stl

For purification of Stl protein, cell pellets were solubilized in 15 ml of buffer A (PBS (pH 7.3), 5 mM MgCl_2_) supplemented with 400 mM NaCl, 2 mM dithiothreitol (DTT), 1% Triton X-100, 2 µg/ml RNase and Dnase. One tablet of Complete ULTRA Tablets, Mini, EDTA-free protease inhibitor preparation was added to every 100 ml of the solution. Cell suspensions were stirred for 10 min, sonicated (4 x 60 s), and centrifuged (16000g for 30 min). Supernatant was diluted in buffer A to contain 200 mM NaCl and loaded on a pre-equlibrated benchtop glutathion-agarose affinity-chromatography column (GE Healthcare). The column was washed with ten volumes of buffer A (200 mM NaCl). After that 80 Cleavage Units thrombin (GE Healthcare) was added to perform on-column cleavage for the removal of GST tag. After overnight cleavage >95% pure Stl protein was eluted from the column. The eluted Stl protein solution was supplemented to contain 400mM NaCl in order to obtain suitable protein stability. The purified preparations appeared as single bands on SDS-PAGE, gel densitometry suggested at least 95% purity**.** The protein preparation was flash-frozen in liquid nitrogen, and stored at 193 K. Before use, aliquots of the enzyme were dialyzed against respective buffers.

### Cloning of tag-free Φ11 dUTPase mutants

The tag free versions of Φ11 dUTPase^F164W^ and Φ11 dUTPase^E158STOP^ proteins were created by QuickChange site-directed mutagenesis (Stratagene) from the pETDuet-Φ11DUT^WT^ construct with the mutagenic primers that were used to create the same Φ11DUT variants with the GST tag (1).

**Purification of tag-free Φ11 dUTPases**

For purification of dUTPase proteins cell pellets were solubilized in buffer B (20 mM HEPES (pH 7.5), 100 mM NaCl, 5 mM MgCl_2_ ,10 mM ß-mercaptoethanol) supplemented with 2 µg/ml RNase and Dnase. One tablet of Complete ULTRA Tablets, Mini, EDTA-free protease inhibitor preparation was added to every 100 ml of the solution. Cell suspensions were stirred for 10 min, sonicated (4 x 60 s), and centrifuged (16000g for 30 min). Supernatants were directly loaded on a Q-Sepharose column (5ml) equilibrated in buffer B (supplemented with 0.1 mM PMSF (phenylmethylsulfonyl fluoride)) and developed using 50 ml of a linear gradient up to 1 M NaCl. dUTPase appeared at 0.3–0.5 M NaCl. A second purification step was performed on a size exclusion column (Superdex 200 10/300 GL, GE Healthcare) in buffer B containing 300 mM NaCl and 0.1 mM PMSF with a molecular weight separation range between 10 000-600 000. AKTA Purifier (GE Healthcare) system, with Unicorn software (GE Healtcare) was used for size exclusion chromatography, absorbance of the eluates were monitored at 280 and 260 nm. The purified preparations appeared as single bands on SDS-PAGE, gel densitometry suggested at least 95% purity**.** Enzyme stocks were concentrated on Millipore centrifugal filters (10 kDa cutoff) to a final concentration, flash-frozen in liquid nitrogen, and stored at 193 K. Before use, aliquots of the enzyme were dialyzed against respective buffers.

### Protein quantification

Protein concentration was measured by Bradford’s assay or spectrophotometrically using A ^0.1%^ _280_ values 1.051, 0.786, 1.083 and 0.84 ml * mg^-1^* cm^-1^ for Stl, Φ11dUTPase^WT^, Φ11dUTPase^F164W^, Φ11dUTPase^E158STOP^ respectively, as calculated from amino acid composition (http://web.expasy.org/protparam/).

### Mass spectrometry (MS)

A commercial Waters QTOF Premier instrument equipped with an electrospray ionization source was used in positive ion mode. The ions were generated from 20 mM NH_4_HCO_3_ buffer solution (pH = 8.0) containing a total protein concentration of 40 μM. These conditions allow transfer of the native protein complexes into the gas phase (2, 3).The capillary voltage was 2600 V, the sampling cone voltage was 128 V and the temperature of the source was kept at 363 K. Mass spectra were recorded in the mass range of 1500 – 6000 m/z.

### Analytic gel filtration

Analytical gel filtration was performed as described in the “Purification of tag-free Φ11 dUTPases” part. Calibration curve was plotted after running successively on the column proteins of well-known molecular weights in order to determine the native oligomerization state of Stl and Φ11 dUTPase, and to determine the complexation of the above mentioned proteins.

### Quartz Crystal Microbalance (QCM) measurements

For the immobilization of Stl LNB-carboxyl chips were prewetted with MilliQ water prior to immobilization, inserted in the Attana A100 QCM biosensor instrument (Attana AB, Stockholm, Sweden), and left to stabilize. Immobilization was carried out at a flow rate of 10 µl/min in HBS-T buffer (10 mM HEPES, 150 mM NaCl, 0.005% Tween 20, pH 7.4) at 25°C. Thereafter, 0.4 mM N-(3-dimethylaminopropyl)-N’-ethylcarbodiimide hydrochloride and 0.1 mM Sulfo-NHS were mixed at 1:1 ratio and this solution was injected immediately with 300 s of contact time to activate the chip’s surface. After rinsing the injection loop with MilliQ water the ligand solution containing 20 µg/ml of purified Stl in 10 mM CH_3_COONa, pH 4.0, was loaded to the chip for 300 s. The injection loop was washed again with MilliQ water, and finally, the remaining activated carboxyl groups on the chip’s surface were neutralized by the injection of 1 M ethanolamine, pH 8.5 for 300 s.

After each measurement the chip’s surface was regenerated by an injection of 50 mM NaOH for 40 s to remove any remaining analyte. At least three independent experiments were performed.

## Steady-state fluorescent measurements

Measurements were done in Greiner 96 Well Black Non-Binding Microplates. Tryptophan residues were excited at 295 nm, emission spectra were recorded between 320-400 nm. For titration, the fluorescence intensities were recorded at 350 nm. Excitation and emission slits were 1nm. Fluorescence was measured in top detection mode, measurement height was optimized for every plate. Additional fluorescence or inner filter effect imposed on the measured intensities during titration experiments were corrected by subtracting the intensity of the assay buffer. The background fluorescence of Stl (Trp:0; Tyr: 24; Phe 6) was subtracted in every case where Stl was present (in these cases buffer was not subtracted).

### Fluorescence kinetics experiments

Long time courses were recorded in a Jobin Yvon Spex Fluoromax-3 spectrofluorometer at 293 K. After 5 minutes preincubation of 2 µM Φ11dUTPase^F164W^ with 4 µM Stl in assay buffer, dUTP was added in various concentrations (500-2300 µM) manually. Trp fluorescence was excited at 297 nm (at 1 nm slit width) and emission was monitored continuously in time at 347 nm. Time courses were analyzed using Origin 7.5. (OriginLab Corp., Northampton, MA).

### Limited proteolysis

Limited tryptic digestion was carried out at 25 °C, using 4.3 µM Stl, 2.2 µM Φ11dUTPase^WT^, 1mM dUPNPP concentrations and 1:800 (w/w) trypsin:Stl ratio in 1.8 mM KH_2_PO_4_, 10mM Na_2_HPO_4_  buffer , pH 7.3, containing 2.7 mM KCl, 200 mM NaCl and 5mM MgCl_2_. Aliquots were taken at different time points. After stopping the digestion by the addition of 1 mM PMSF, samples were analyzed by SDS-PAGE.

### Production of Stl binding site_183_ for EMSA experiments

The 171mer oligonucleotide, that was used in previously for EMSA experiments (4), and was custom synthetized by Eurofins MWG Operon and cloned into the NotI/NotI sites of the vector pEX-A. The sequence was additionally flanked by an EcoRI site on 5’ and BamHI site on the 3’ end. The binding site was amplified for EMSA experiments yielding a 183mer oligo (Stl binding site_183_). For amplification Stl_oligo_F (5’-GAATTCATTTCAACATTAAATATTG-3’) and Stl_oligo_R (5’-GGATCCTAAATCCTGTCCTTTCAC-3’) primers were used. The PCR product was purified with Quiagen PCR clean up KIT.

Supplemental References

1. Leveles, I., Németh, V., Szabó, J.E., Harmat, V., Nyíri, K., Bendes, Á.Á., Papp-Kádár, V., Zagyva, I., Róna, G., Ozohanics, O., et al. (2013) Structure and enzymatic mechanism of a moonlighting dUTPase. *Acta Crystallogr. D. Biol. Crystallogr.*, **69**, 2298–308.

2. Benesch, J.L.P. and Robinson, C. V (2006) Mass spectrometry of macromolecular assemblies: preservation and dissociation. *Curr. Opin. Struct. Biol.*, **16**, 245–51.

3. Grandori, R., Santambrogio, C., Brocca, S., Invernizzi, G. and Lotti, M. (2009) Electrospray-ionization mass spectrometry as a tool for fast screening of protein structural properties. *Biotechnol. J.*, **4**, 73–87.

4. Tormo-Más, M.A., Mir, I., Shrestha, A., Tallent, S.M., Campoy, S., Lasa, I., Barbé, J., Novick, R.P., Christie, G.E. and Penadés, J.R. (2010) Moonlighting bacteriophage proteins derepress staphylococcal pathogenicity islands. *Nature*, **465**, 779–82.

5. Kuroda, M., Ohta, T., Uchiyama, I., Baba, T., Yuzawa, H., Kobayashi, I., Cui, L., Oguchi, a, Aoki, K., Nagai, Y., et al. (2001) Whole genome sequencing of meticillin-resistant Staphylococcus aureus. *Lancet*, **357**, 1225–40.

6. Mwangi, M.M., Wu, S.W., Zhou, Y., Sieradzki, K., de Lencastre, H., Richardson, P., Bruce, D., Rubin, E., Myers, E., Siggia, E.D., et al. (2007) Tracking the in vivo evolution of multidrug resistance in Staphylococcus aureus by whole-genome sequencing. *Proc. Natl. Acad. Sci. U. S. A.*, **104**, 9451–6.

7. Nübel, U., Dordel, J., Kurt, K., Strommenger, B., Westh, H., Shukla, S.K., Zemlicková, H., Leblois, R., Wirth, T., Jombart, T., et al. (2010) A timescale for evolution, population expansion, and spatial spread of an emerging clone of methicillin-resistant Staphylococcus aureus. *PLoS Pathog.*, **6**, e1000855.

8. Holden, M.T.G., Lindsay, J. a, Corton, C., Quail, M. a, Cockfield, J.D., Pathak, S., Batra, R., Parkhill, J., Bentley, S.D. and Edgeworth, J.D. (2010) Genome sequence of a recently emerged, highly transmissible, multi-antibiotic- and antiseptic-resistant variant of methicillin-resistant Staphylococcus aureus, sequence type 239 (TW). *J. Bacteriol.*, **192**, 888–92.

9. Golding, G.R., Bryden, L., Levett, P.N., McDonald, R.R., Wong, A., Graham, M.R., Tyler, S., Van Domselaar, G., Mabon, P., Kent, H., et al. (2012) whole-genome sequence of livestock-associated st398 methicillin-resistant staphylococcus aureus Isolated from Humans in Canada. *J. Bacteriol.*, **194**, 6627–8.

10. Larner-Svensson et al. (2013) Complete genome sequence of Staphylococcus aureus strain M1, a unique t024-ST8-IVa Danish methicillin-resistant S. aureus clone. *Genome Announc.*, **1**, e00336–13.

11. Herron-Olson, L., Fitzgerald, J.R., Musser, J.M. and Kapur, V. (2007) Molecular correlates of host specialization in Staphylococcus aureus. *PLoS One*, **2**, e1120.

12. Zhou, Y., Liang, Y., Lynch, K.H., Dennis, J.J. and Wishart, D.S. (2011) PHAST: a fast phage search tool. *Nucleic Acids Res.*, **39**, W347–52.

13. Vogel, V., Falquet, L., Calderon-Copete, S.P., Basset, P. and Blanc, D.S. (2012) Short term evolution of a highly transmissible methicillin-resistant Staphylococcus aureus clone (ST228) in a tertiary care hospital. *PLoS One*, **7**, e38969.

14. Stegger, M., Price, L.B., Larsen, A.R., Gillece, J.D., Waters, A.E., Skov, R. and Andersen, P.S. (2012) Genome sequence of Staphylococcus aureus strain 11819-97, an ST80-IV European community-acquired methicillin-resistant isolate. *J. Bacteriol.*, **194**, 1625–6.

15. Uhlemann, A.-C., Porcella, S.F., Trivedi, S., Sullivan, S.B., Hafer, C., Kennedy, A.D., Barbian, K.D., McCarthy, A.J., Street, C., Hirschberg, D.L., et al. (2012) Identification of a highly transmissible animal-independent Staphylococcus aureus ST398 clone with distinct genomic and cell adhesion properties. *MBio*, **3**, 1–9.

16. Gill, S.R., Fouts, D.E., Archer, G.L., Mongodin, E.F., Deboy, R.T., Ravel, J., Paulsen, I.T., Kolonay, J.F., Brinkac, L., Beanan, M., et al. (2005) Insights on evolution of virulence and resistance from the complete genome analysis of an early methicillin-resistant Staphylococcus aureus strain and a biofilm-producing methicillin-resistant Staphylococcus epidermidis strain. *J. Bacteriol.*, **187**, 2426–38.

17. Guinane, C.M., Ben Zakour, N.L., Tormo-Mas, M. a, Weinert, L. a, Lowder, B. V, Cartwright, R. a, Smyth, D.S., Smyth, C.J., Lindsay, J. a, Gould, K. a, et al. (2010) Evolutionary genomics of Staphylococcus aureus reveals insights into the origin and molecular basis of ruminant host adaptation. *Genome Biol. Evol.*, **2**, 454–66.

18. Lowder, B. V, Guinane, C.M., Ben Zakour, N.L., Weinert, L. a, Conway-Morris, A., Cartwright, R. a, Simpson, a J., Rambaut, A., Nübel, U. and Fitzgerald, J.R. (2009) Recent human-to-poultry host jump, adaptation, and pandemic spread of Staphylococcus aureus. *Proc. Natl. Acad. Sci. U. S. A.*, **106**, 19545–50.

19. Köser, C.U., Holden, M.T.G., Ellington, M.J., Cartwright, E.J.P., Brown, N.M., Ogilvy-Stuart, A.L., Hsu, L.Y., Chewapreecha, C., Croucher, N.J., Harris, S.R., et al. (2012) Rapid whole-genome sequencing for investigation of a neonatal MRSA outbreak. *N. Engl. J. Med.*, **366**, 2267–75.

20. Chua, K.Y.L., Seemann, T., Harrison, P.F., Monagle, S., Korman, T.M., Johnson, P.D.R., Coombs, G.W., Howden, B.O., Davies, J.K., Howden, B.P., et al. (2011) The dominant Australian community-acquired methicillin-resistant Staphylococcus aureus clone ST93-IV [2B] is highly virulent and genetically distinct. *PLoS One*, **6**, e25887.

21. Harrison, E.M., Paterson, G.K., Holden, M.T.G., Larsen, J., Stegger, M., Larsen, A.R., Petersen, A., Skov, R.L., Christensen, J.M., Bak Zeuthen, A., et al. (2013) Whole genome sequencing identifies zoonotic transmission of MRSA isolates with the novel mecA homologue mecC. *EMBO Mol. Med.*, **5**, 509–15.

22. Huang, T.-W., Chen, F.-J., Miu, W.-C., Liao, T.-L., Lin, A.-C., Huang, I.-W., Wu, K.-M., Tsai, S.-F., Chen, Y.-T. and Lauderdale, T.-L.Y. (2012) Complete genome sequence of Staphylococcus aureus M013, a pvl-positive, ST59-SCCmec type V strain isolated in Taiwan. *J. Bacteriol.*, **194**, 1256–7.

23. Holden, M.T.G., Feil, E.J., Lindsay, J. a, Peacock, S.J., Day, N.P.J., Enright, M.C., Foster, T.J., Moore, C.E., Hurst, L., Atkin, R., et al. (2004) Complete genomes of two clinical Staphylococcus aureus strains: evidence for the rapid evolution of virulence and drug resistance. *Proc. Natl. Acad. Sci. U. S. A.*, **101**, 9786–91.

24. Holt, D.C., Holden, M.T.G., Tong, S.Y.C., Castillo-Ramirez, S., Clarke, L., Quail, M. a, Currie, B.J., Parkhill, J., Bentley, S.D., Feil, E.J., et al. (2011) A very early-branching Staphylococcus aureus lineage lacking the carotenoid pigment staphyloxanthin. *Genome Biol. Evol.*, **3**, 881–95.

25. Baba, T., Takeuchi, F., Kuroda, M., Yuzawa, H., Aoki, K., Oguchi, A., Nagai, Y., Iwama, N., Asano, K., Naimi, T., et al. (2002) Genome and virulence determinants of high virulence community-acquired MRSA. *Lancet*, **359**, 1819–27.

26. Neoh, H., Cui, L., Yuzawa, H., Takeuchi, F., Matsuo, M. and Hiramatsu, K. (2008) Mutated response regulator graR is responsible for phenotypic conversion of Staphylococcus aureus from heterogeneous vancomycin-intermediate resistance to vancomycin-intermediate resistance. *Antimicrob. Agents Chemother.*, **52**, 45–53.

27. Iandolo, J.J., Worrell, V., Groicher, K.H., Qian, Y., Tian, R., Kenton, S., Dorman, A., Ji, H., Lin, S., Loh, P., et al. (2002) Comparative analysis of the genomes of the temperate bacteriophages phi 11, phi 12 and phi 13 of Staphylococcus aureus 8325. *Gene*, **289**, 109–18.

28. Schijffelen, M.J., Boel, C.H.E., van Strijp, J. a G. and Fluit, A.C. (2010) Whole genome analysis of a livestock-associated methicillin-resistant Staphylococcus aureus ST398 isolate from a case of human endocarditis. *BMC Genomics*, **11**, 376.

29. Hung, W.-C., Takano, T., Higuchi, W., Iwao, Y., Khokhlova, O., Teng, L.-J. and Yamamoto, T. (2012) Comparative genomics of community-acquired ST59 methicillin-resistant Staphylococcus aureus in Taiwan: novel mobile resistance structures with IS1216V. *PLoS One*, **7**, e46987.

30. Li, Y., Cao, B., Zhang, Y., Zhou, J., Yang, B. and Wang, L. (2011) Complete genome sequence of Staphylococcus aureus T0131, an ST239-MRSA-SCCmec type III clone isolated in China. *J. Bacteriol.*, **193**, 3411–2.

31. Diep, B.A., Gill, S.R., Chang, R.F., Phan, T.H., Chen, J.H., Davidson, M.G., Lin, F., Lin, J., Carleton, H.A., Mongodin, E.F., et al. (2006) Complete genome sequence of USA300, an epidemic clone of community-acquired meticillin-resistant Staphylococcus aureus. *Lancet*, **367**, 731–9.

32. Highlander, S.K., Hultén, K.G., Qin, X., Jiang, H., Yerrapragada, S., Mason, E.O., Shang, Y., Williams, T.M., Fortunov, R.M., Liu, Y., et al. (2007) Subtle genetic changes enhance virulence of methicillin resistant and sensitive Staphylococcus aureus. *BMC Microbiol.*, **7**, 99.

33. Howden, B.P., Seemann, T., Harrison, P.F., McEvoy, C.R., Stanton, J.-A.L., Rand, C.J., Mason, C.W., Jensen, S.O., Firth, N., Davies, J.K., et al. (2010) Complete genome sequence of Staphylococcus aureus strain JKD6008, an ST239 clone of methicillin-resistant Staphylococcus aureus with intermediate-level vancomycin resistance. *J. Bacteriol.*, **192**, 5848–9.

34. Baba, T., Bae, T., Schneewind, O., Takeuchi, F. and Hiramatsu, K. (2008) Genome sequence of Staphylococcus aureus strain Newman and comparative analysis of staphylococcal genomes: polymorphism and evolution of two major pathogenicity islands. *J. Bacteriol.*, **190**, 300–10.

35. Fraunholz, M., Bernhardt, J., Schuldes, J., Daniel, R., Hecker, M. and Sinha, B. (2013) Complete Genome Sequence of Staphylococcus aureus 6850, a Highly Cytotoxic and Clinically Virulent Methicillin-Sensitive Strain with Distant Relatedness to Prototype Strains. *Genome Announc.*, **1**.

36. Chen, C.-J., Unger, C., Hoffmann, W., Lindsay, J.A., Huang, Y.-C. and Götz, F. (2013) Characterization and comparison of 2 distinct epidemic community-associated methicillin-resistant Staphylococcus aureus clones of ST59 lineage. *PLoS One*, **8**, e63210.

37. Tóth, J., Varga, B., Kovács, M., Málnási-Csizmadia, A. and Vértessy, B.G. (2007) Kinetic mechanism of human dUTPase, an essential nucleotide pyrophosphatase enzyme. *J. Biol. Chem.*, **282**, 33572–82.

38. John, J., Sohmen, R., Feuerstein, J., Linke, R., Wittinghofer, A. and Goody, R.S. (1990) Kinetics of interaction of nucleotides with nucleotide-free H-ras p21. *Biochemistry*, **29**, 6058–65.

39. Rensland, H., John, J., Linke, R., Simon, I., Schlichting, I., Wittinghofer, A. and Goody, R.S. (1995) Substrate and product structural requirements for binding of nucleotides to H-ras p21: the mechanism of discrimination between guanosine and adenosine nucleotides. *Biochemistry*, **34**, 593–9.

40. Haller, M. (1997) Nucleotide Hydrolysis-dependent Conformational Changes in p21ras as Studied Using ESR Spectroscopy. *J. Biol. Chem.*, **272**, 30103–30107.

41. Gideon, P., John, J., Frech, M., Lautwein, A., Clark, R., Scheffler, J.E. and Wittinghofer, A. (1992) Mutational and kinetic analyses of the GTPase-activating protein (GAP)-p21 interaction: the C-terminal domain of GAP is not sufficient for full activity. *Mol. Cell. Biol.*, **12**, 2050–6.

42. Herrmann, C., Martin, G.A. and Wittinghofer, A. (1995) Quantitative analysis of the complex between p21ras and the Ras-binding domain of the human Raf-1 protein kinase. *J. Biol. Chem.*, **270**, 2901–5.

43. Sydor, J.R., Engelhard, M., Wittinghofer, a, Goody, R.S. and Herrmann, C. (1998) Transient kinetic studies on the interaction of Ras and the Ras-binding domain of c-Raf-1 reveal rapid equilibration of the complex. *Biochemistry*, **37**, 14292–9.

44. Tormo-Más, M.Á., Donderis, J., García-Caballer, M., Alt, A., Mir-Sanchis, I., Marina, A. and Penadés, J.R. (2013) Phage dUTPases control transfer of virulence genes by a proto-oncogenic G protein-like mechanism. *Mol. Cell*, **49**, 947–58.

45. Pécsi, I., Szabó, J.E., Adams, S.D., Simon, I., Sellers, J.R., Vértessy, B.G. and Tóth, J. (2011) Nucleotide pyrophosphatase employs a P-loop-like motif to enhance catalytic power and NDP/NTP discrimination. *Proc. Natl. Acad. Sci. U. S. A.*, **108**, 14437–42.

**Supplemental Tables**

**Supplemental Table I.** Rate constants of the potential conformational change observed by QCM

|  | k_2_ (s^-1^) | k_-2_ (s^-1^) |
| --- | --- | --- |
| Φ11 dUTPase^WT^ | 0.0105 ± 0.0021 | 0.0012 ± 0.0003 |
| Φ11 dUTPase^F164W^ | 0.0083 ± 0.0004 | 0.0011 ± 0.0001 |
| Φ11 dUTPase^F164W^, dUTP | 0.0120 ± 0.0018 | 0.0046 ± 0.0011 |
| Φ11 dUTPase^F164W^, dUMP | 0.0089 ± 0.0008 | 0.0016 ± 0.0002 |

**Supplemental Table II.** Occurrence of dUTPases in *Staphylococcus aureus* strains

| **Genome sequence** | **Organism**  **(NCBI Genome)** | **Genomic dUTPase^1^** | **Phage dUTPases** | | | |
| --- | --- | --- | --- | --- | --- | --- |
|  |  |  | **Dimer^1^** | **Trimer^1^** | **Prophage carrying dUTPase^2^** | **Reference for prophage detection** |
| NC_002745.2 | N315 | − | 1124674 | − | ΦN315 | (5) |
| NC_009632.1 | JH1 | − | 5317510 | − | Phage 92 –like | (6) |
|  |  |  | − | 5317603 | ΦSaST5-K | (6, 7) |
|  |  |  | 5317432 | − | ΦSa2_USA_-like | (6) |
|  |  |  | 5317353 | − | ΦN315 -like |  |
| NC_017331.1 | TW20 | − | 12862042 | − | ΦSa1_TW20_ | (8) |
|  |  |  | 12863174 | − | ΦSa3_TW20_ |  |
| NC_017340.1 | 04-02981 | − | − | 12865016 | ΦSaST5-K | (7) |
|  |  |  | 12864800 | − | ΦN315 |  |
| NC_018608.1 | 08BA02176 | − | − | − | − | (9) |
| NC_021059 | M1 | − | 15299711 | − | ΦSa2_USA_ –like | (10) |
|  |  |  | 15300291 | − | ΦSa3_USA_ –like |  |
| NC_007622.1 | RF122 | − | 3795196 | − | ΦSaBov | (11) |
| NC_020566.1 | ST228 _isolate 16125_ | − | 14837587 | − | P954-like_(100/ 1984860)_ | (12, 13) |
| NC_020568.1 | ST228 _isolate 18583_ | − | 14839621 | − | ΦNM3-like_(60/1984723)_ |  |
| NC_020564.1 | ST228 _isolate 10497_ | − | 14835590 | − | ΦNM3-like_(70/1984906)_ |  |
| NC_020537.1 | ST228 _isolate 18412_ | − | 14817523 | − | P954-like_(70/1984670)_ |  |
| NC_020536.1 | ST228 _isolate 18341_ | − | 14815488 | − | P954-like_(90/1984905)_ |  |
| NC_020533.1 | ST228 _isolate 16035_ | − | 14813492 | − | P954-like_(90/1984851)_ |  |
| NC_020532.1 | ST228 _isolate 15532_ | − | 14811453 | − | P954-like_(60/1985219)_ |  |
| NC_020529.1 | ST228 _isolate 10388_ | − | 14809415 | − | P954-like_(70/1984919)_ |  |
| NC_017351.1 | 11819-97 | − | − | 12424268 | ΦSa2 | (14) |
|  |  |  | 12424797 | − | ΦSa3, |  |
|  |  |  | 12423395 | − | Φ37-like |  |
| NC_017673.1 | 71193 | − | − | 12731126 | Φ3 | (15) |
| NC_002951.2 | Col | − | − | 3236924 | ΦCol | (16) |
| NC_017343.1 | ECT-R2 | − | 12336428 | − | ΦN315-ike_(136/1950872)_ | (12) |
| NC_017337.1 | ED-133 | − | 12324986 | − | ΦSaov1 | (17) |
|  |  |  | − | 12324459 | ΦSaov2 |  |
|  |  |  | − | 12323829 | ΦSaov3 |  |
| NC_013450.1 | ED-98 | − | − | 8613603 | ΦAv1 | (18) |
|  |  |  | 8614777 | − | ΦAvß |  |
| NC_017763.1 | HO 5096 0412 | − | − | 12799907 | ΦSa2 | (19) |
|  |  |  | − | 12801001 | ΦSa3 |  |
| NC_009487.1 | JH9 | − | 5168136 | − | Phage 92 –like | (6) |
|  |  |  | − | 5169631 | ΦSaST5-K | (6, 7) |
|  |  |  | 5168479 | − | ΦSa2_USA_-like | (6) |
|  |  |  | 5168299 | − | ΦN315 -like |  |
| NC_017338.1 | JKD6159 | − | 12327664 | − | ΦSa2 | (20) |
|  |  |  | 12326670 | − | ΦSa3 |  |
| NC_017349.1 | LGA251 | − | − | 12907879 | ΦSa8 | (21) |
| NC_016928.1 | M013 | − | 11862817 | − | ΦPVL | (22) |
| NC_002952.2 | MRSA252 | − | 2860609 | − | ΦSa2 | (23) |
|  |  |  | − | 2861519 | ΦSa3 |  |
| NC_016941.1 | MSHR1132 | − | − | 11931868 | ΦSa3 | (24) |
| NC_002953.3 | MSSA476 | − | 2863935 | − | ΦSa4 | (23) |
| NC_003923.1 | MW2 | − | 1003527 | − | ΦSa2_MW_ | (25) |
| NC_009782.1 | Mu3 | − | − | 5559151 | Mu50A_Mu3_ | (5, 26) |
|  |  |  | 5560392 | − | Mu50B _Mu3_ |  |
| NC_002758.2 | Mu50 | − | − | 1121986 | Mu50A | (5) |
|  |  |  | 1120851 | − | Mu50B |  |
| NC_007795.1 | NCT8325 | − | − | 3919744 | Φ11 | (27) |
|  |  |  | − | 3920595 | Φ12 |  |
|  |  |  | 3919570 | − | Φ13 |  |
| NC_017333.1 | ST398 | − | 12322852 | − | ΦSa2_S0385_ | (28, 29) |
|  |  |  | 12323353 | − | ΦSa6_S0385_ |  |
| NC_017347.1 | T0131 | − | 12338028 | − | ΦNM3-like_(100/2058773)_ | (12, 30) |
| NC_017342.1 | TCH60 | − | − | 12333594 | Φ7401PVL-like_(150/1737832)_ | (12) |
| NC_007793.1 | USA300_FPR3757 | − | 3913324 | − | ΦSa2_USA_ | (31) |
|  |  |  | − | 3913209 | ΦSa3_USA_ |  |
| NC_010079.1 | USA300_TCH1516 | − | 5776354 | − | ΦSLT_USA300_ | (32) |
|  |  |  | − | 5776354 | Φß_USA300_ |  |
| NC_016912.1 | VC40 | − | − | − | − | − |
| NC_017341.1 | JKD6008 | − | 12331190 | − | Phage 92 –like_(60/1964562)_ | (12, 33) |
|  |  |  | 12331352 | − | ΦNM3_(60/2113993)_ |  |
| NC_009641.1 | Newman | − | 5331083 | − | ΦNM1 | (34) |
|  |  |  | 5330616 | − | ΦNM2 |  |
|  |  |  | − | 5331168 | ΦNM3 |  |
|  |  |  | 5330123 | − | ΦNM4 |  |
| NC_022222.1^3^ | 6850 | − | − | − | − | (12, 35) |
| NC_022113.1^3^ | 55/2053 | − | 16643574 | − | ΦPVL108-like_(90/1971076)_ | (12) |
|  |  |  | − | 16643031 | ΦNM3-like_(130/1457369)_ |  |
| NC_021670.1^3^ | Bmb9393 | − | 16042074 | − | P954-like_(80/2165809)_ | (12) |
|  |  |  | − | 16041143 | StauST398_2-like_(150/1165187)_ |  |
|  |  |  | − | 16041551 | ΦMR1-like_(1130/1632359)_ |  |
| NC_021554.1^3^ | CA-347 | − | 16168865 | − | Staphy_2-like_(117/2068311)_ | (12) |
|  |  |  | 16169566 | − | ΦNM3-like_(150/1517475)_ |  |
| NC_022226.1^3^ | CN1 | − | 16743935 | − | ΦNM3-like_(113/1973210)_ | (12) |
| NC_022443.1^3^ | SA40 | − | − | − | − | (12, 36) |
| NC_022442.1^3^ | SA957 | − | 17054050 | − | Φ5967PVL-like_(146/1523591)_ | (12, 36) |
| NC_022604.1^3^ | Z172 | − | 17370004 | − | ΦNM3-like_(108/2105565)_ | (12) |
|  |  |  | 17369075 | − | ΦMR11-like_(100/1151302)_ |  |

^1^ Gene ID of the hits is given in the indicated columns.

^2^ Only the prophages that carry a dUTPase are indicated. In case of PHAST detection the PHAST score of the appropriate hit and the starting site of the prophage region are indicated as subscript (score/starting site). Meaning of PHAST scores: >90 indicates intact and inducible prophage, 60-90 indicates that the inducibility of the prophage is quiestionable, <60 indicates that the prophage is probable not activable anymore.

^3^ Provisional REFSEQ

### Supplemental Table III. Kinetic comparison of dUTPase and G protein based switches.

|  | **dUTPase*** | **p21^H-Ras^ **** |
| --- | --- | --- |
| **Properties of nucleotide hydrolase reaction** | | |
| d(NTP) k_on_ (µM^-1^s^-1^) | 1.2 * 10^2^ (37) | 2.1 (38) |
| d(NTP) k_off_ (s^-1^) | 1.0 * 10^2^ (37) | 2.9 * 10^-5^ (39) |
| K_d_ (µM) (k_off_ / k_on_) | 8.3 * 10^-1^ (37) | 1.4 * 10^-5^ |
| k_on_ product nucl. (µM^-1^s^-1^) | 3.1 * 10^1^ (37) | 8.4 * 10^-1^ (38) |
| k_off_ product nucl. (s^-1^) | 1.0 * 10^3^ (37) | 1.8 * 10^-5^ (38) |
| K_d_ product nucl.(µM) (k_off_ / k_on_) | 3.2 * 10^1^ (37) | 2.1 * 10^-5^ (38) |
| k_cat_ (s^-1^) | 6.4 (37) | 5.5 * 10^-3^ (40) |
| p120-GAP activated k_cat_ (s^-1^) | NA | 10^5^ stimulation (41) |
| **Interaction with the effector** | | |
|  | Stl | Ras Binding domain of Raf |
| Effect on activity | total inhibition | none (42) |
| k_on_ (µM^-1^s^-1^) | 4.1 * 10^-1^ (This work)^#^ | NA |
| k_off_ (s^-1^) | 2.8 * 10^-3^ (This work) ^#^ | NA |
| K_d_ (µM) (k_off_ / k_on_) | 6.8 * 10^-2^ (This work) ^##^ | NA |
| protein.d(NTP) k_on_ (µM^-1^s^-1^) | NA | 4.5 * 10^1^ (43) |
| protein.d(NTP) k_off_ (s^-1^) | NA | 7.4 (43) |
| protein.d(NTP) K_d_ (µM) (k_off_ / k_on_) | NA | 1.6 * 10^-1^ (43) |
| protein.product nucl. K_d_ (µM) (K_eqv_). | 9.2 * 10^-2^ (This work) ^##^ | 3.2 * 10^1^ (43) |

* 20°C; ** 25°C; ^#^ From stopped-flow experiments, cf. Table I. ^##^ From QCM experiment

### Supplemental Table IV. Our model is consistent with previous *in vivo* results

| Protein | K_d_ (µM) | Activity (%)* | Probable state *in vivo* | SAPI induction | Consistent with our model? |
| --- | --- | --- | --- | --- | --- |
| Φ11 dUTPase^WT^ | 0.8 ^2^ | 157 ^3^ | dUTP bound until  dUTP is converted into dUMP | Yes (better than 80α) | YES |
| 80α dUTPase^WT^ | 0.88 ^4^ | 100 ^4^ | dUTP bound until dUTP is converted into dUMP | Yes ^4^ | YES |
| 80α dUTPase^D81A^ | 2.63 ^4^ | 0 ^4^ | dUTP bound | no, only if overexpressed ^4^ | YES |
| 80α dUTPase^D81N^ | 2.64 ^4^ | 0 ^4^ | dUTP bound | No ^4^ | YES |
| 80α dUTPase^Y84A^ | 3.37 ^4^ | 24 ^4^ | dUTP bound until dUTP is converted into dUMP | Low ^4^ | YES |
| 80α dUTPase^Y84F^ | 0.88 ^4^ | 77 ^4^ | dUTP bound until dUTP is converted into dUMP | Yes ^4^ | YES |
| 80α dUTPase^Y84I^ | >400 ^4^ | 0 ^4^ | substrate free | No ^4^ | not enough information |
| 80α dUTPase^ΔV^ | ND, probably similar to dUDP binding^5^ | 0 ^4^ | dUTP/dUDP bound^5^ | No ^4^ | YES |
| C-C | ND | 0 ^4^ | not enough information | Hyperactive ^4^ | not enough information, |
| 80α dUTPase^D95E^ | ND | 66 ^3^ | dUTP bound until dUTP is converted into dUMP | No ^3^ | not enough information |

* 100% activity relates to the activity of wild type 80α dUTPase, the values of the mutant species are related to this value

^2^ (1)

^3^ (4)

^4^ (44)

^5^ (45)

**Supplemental figures**

### **Supplemental Figure 1:** Φ11 dUTPase^WT^ and Stl form a tight complex with slow kinetics: Supplemental results


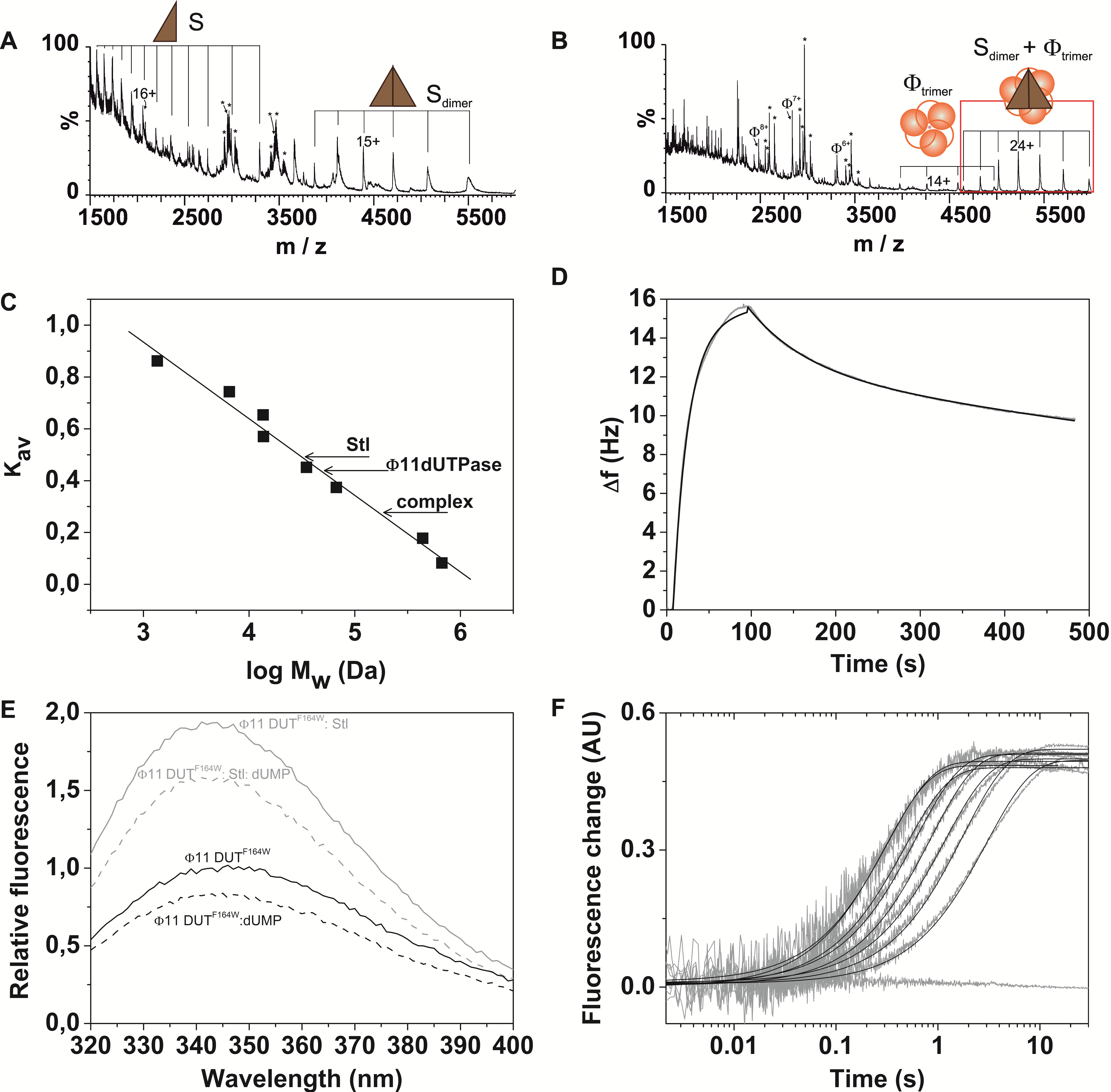


Mass spectra of the Stl protein (**Panel A**) and a 1:1 mixture of the Stl (S) and Φ11 dUTPase^WT^ (Φ) proteins (**Panel B)** measured under native electrospray conditions. Subscripts indicate the oligomerization states of the proteins, while numbers in superscripts and next to the peaks indicate the charge states. The red frame in part B highlights the region of the Φ_3_S_2_ complex, supporting the specific interaction between the proteins under study. Impurities are marked with asterisk. **Panel C** Size-exclusion chromatography indicates stable complex formation between Stl and dUTPase. **Panel D** shows QCM experiments for complex formation between Stl and wild type Φ11 DUT^WT^ (grey line). A solid black line represents the fit of two state reaction model. Rate constants from the fitted model are shown in Table I and Suppl. Table I. **Panel E** shows steady-state fluorescence spectra of 3 µM Φ11DUT^F164W^ enzyme with (dashed line) and without (solid line) 2mM dUMP in the presence (grey lines) and absence (black lines) of 3 µM Stl. Data were normalized to the emission peak (346 nm) of the free Φ11DUT^F164W^. The maximum relative fluorescence of the complexes were: 0.84 at 345 nm for Φ11DUT^F164W^: dUMP, 1.94 at 341 nm for Φ11DUT^F164W^: Stl and 1.53 at 341 nm for Φ11DUT^F164W^: Stl: dUMP. **Panel F** shows the fluorescence time courses observed upon the binding of Stl (1-8 µM) to 0.25 µM Φ11DUT^F164W^. The curves are shown from 0.002 s (after the deadtime). Smooth lines are single exponential fit to the experimental curves. No further resolvable fluorescence phases were observed.

### Supplemental Figure 2: dUTPase:Stl complex formation eliminates the physiological function of both proteins: Supplemental results





**Panel A** Fluorescence equilibrium titration of Φ11DUT^F164W^ (2 µM):Stl (3.5 µM) complex with dUPNPP. **Panel B**. shows QCM measurements of Φ11DUT^F164W^ in the absence and presence of dUMP and dUTP. **Panel C.** Limited proteolysis of Stl and Φ11DUT^WT^ complex in absence and in presence of dUPNPP. Trypsin was used for digestion of Stl and Φ11DUT^WT^ proteins. Black arrows indicate the position of intact Stl or Φ11DUT^WT^. First lane of each gel corresponds to molecular mass marker with 35 and 25 kDa bands. The following lanes represent the tryptic digestion at different time points (0 min, 5 min, 15 min, 30 min, 45 min and 60 min, respectively). **Panel D** shows the interaction of 2.3 mM dUTP with premixed Φ11DUT^F164W^(2 µM):Stl(4 µM) complex (red curve). Single exponential fitting to the first phase with fluorescence decreasing (represented as smooth black line) yield observed rate constant 0.00303 ± 0.00008 s^-1^.

**Supplemental Figure 3: dUTPase eliminates Stl binding to its cognate DNA element independently of the presence of its C-terminal segment**

**
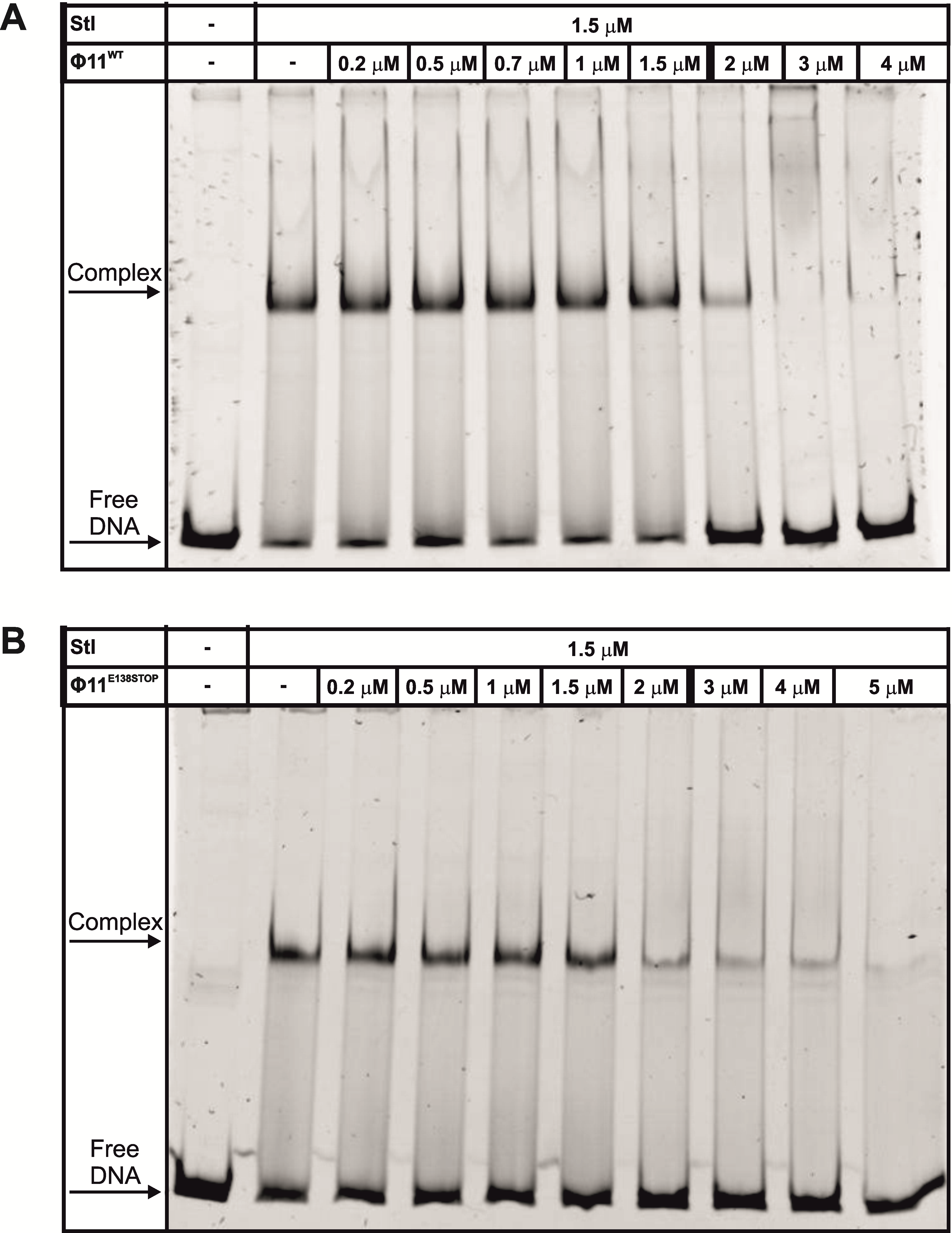
**

**Panel A** Φ11 DUT^WT^ prevents Stl from binding to the Stl binding site_183_ shown in EMSA. 1.5 µM Stl was titrated with increasing amounts of Φ11 DUT^WT^ (0.2, 0.5, 0.7 1, 1.5, 2,3,4 µM). **Panel B** Φ11 DUT^E158STOP^ prevents Stl from binding to the Stl binding site_183_ shown in EMSA. 1.5 µM Stl was titrated with increasing amounts of Φ11 DUT^E158STOP^ (0.2, 0.5, 1, 1.5, 2, 3, 4, 5 µM). Arrows on the left show the positions of free oligonucleotide and Stl bound oligonucleotide.
